# Successful control with carbamazepine of family with paroxysmal kinesigenic dyskinesia of PRRT2 mutation

**DOI:** 10.7603/s40681-014-0015-0

**Published:** 2014-05-08

**Authors:** I-Ching Chou, Sheng-Shing Lin, Wei-De Lin, Chung-Hsing Wang, Yu-Tzu Chang, Fuu-Jen Tsai, Chang-Hai Tsai

**Affiliations:** 1 Department of Pediatrics, Children’s Hospital, China Medical University Hospital, Taichung, Taiwan; 2 Graduate Institute of Integrated Medicine, College of Chinese Medicine, China Medical University, Taichung, Taiwan; 3 Department of Medical Research, China Medical University and Hospital, Taichung, Taiwan; 4 School of Post Baccalaureate Chinese Medicine, China Medical University, Taichung, Taiwan; 5 Asia University, Taichung, Taiwan; 6 Department of Pediatrics, China Medical University Hospital, 2 Yuh-Der Road, Taichung 404, Taichung, Taiwan

**Keywords:** Carbamazepin, Paroxysmal dyskinesia, *PRRT2*, Mutation

## Abstract

Paroxysmal kinesigenic dyskinesia (PKD), a rare paroxysmal movement disorder often misdiagnosed as epilepsy, is characterized by recurrent, brief dyskinesia attacks triggered by sudden voluntary movement. Pathophysiological mechanism of PKD remains not well understood. Ion channelopathy has been suggested, since the disease responds well to ion channel blockers. Mutations in proline-rich transmembrane protein 2 (*PRRT2*) were recently identified in patients with familial PKD. To extend these genetic reports, we studied a family with clinical manifestations of familial PKD responding well to low dose carbamazepine. Therapeutic dose ranged from 1.5 to 2.0 mg/ kg/day, below that in seizure control. One insertion mutation c.649_650insC (p.P217fsX7) was identified in three patients of the family. This study avers *PRRT2*’s high sensitivity for PKD phenotype. Identification of genes underlying pathogenesis will enhance diagnosis and treatment. Function of *PRRT2* and its role in PKD warrant further investigation.

## 1. Introduction

Paroxysmal kinesigenic dyskinesia (PKD, OMIM 128000) is a rare paroxysmal movement disorder, often misdiagnosed as epilepsy and characterized by recurrent, brief attacks of dyskinesia triggered by sudden voluntary movement. Onset is around early teenage [1]. Sudden movement after a prolonged rest period is the most common precipitating factor, duration of attack usually brief, lasting seconds up to five minutes [2-5]. They can occur many times daily, but frequency and severity of attack seem to decrease with age. Brain imaging and electroencephalography (EEG) of such patients are usually normal [1, 6] Pathophysiological mechanism of PKD remains not well understood, but studies suggest it as a type of reflex epilepsy [7-9]. Ion channelopathy has been cited; this disease responds well to ion channel blockers [10]. Mutations of proline-rich transmembrane protein 2 (*PRRT2*) have recently been identified in cases of familial PKD [11-15]. *PRRT2* protein, expressed strongly in a developing nervous system and localized to axons, interacts with synaptic protein SNAP 25, and may play a role in synaptic regulation [11, 14]. We report a family with clinical manifestations of familial PKD responding to low-dose CBZ. One insertion mutation c.649_650insC (p.P217fsX7) was identified in three patients of the family.

## 2. Patients and Methods

### 2.1. Clinical Report

A twelve-year-old male had two-year history of paroxysmal movement disorder induced by sudden movement or brief exercise; birth, delivery, and development were unremarkable, without significant medical history. Attacks were first evident at age 10, after sudden arm movements; these entailed dystonic flexion and greater internal rotation of left arm and left leg, as well as finger posturing. He was fully aware of his surroundings during the attacks (4-5 times/day), lasting 10-20 (rarely 30) seconds. General physical and neurologic examinations proved normal. Magnetic resonance imaging of the brain, interictal electroencephalogram, and laboratory workup yielded unremarkable results; carbamazepine was prescribed upon diagnosis of paroxysmal kinesigenic dyskinesia. At 200 mg daily (10 mg/kg/day), the patient attained complete resolution of signs that has persisted for the past seven years, no side effects from medication. His father and brother, both diagnosed as PKD, had the same symptoms (Fig. A) ; Table 1 summarizes clinical manifestations. Both were prescribed CBZ as monotherapy (dosage 200 mg/day) to attain total resolution for the past seven years.

**Table 1:** Clinical Summary of Three Patients with Paroxysmal Kinesigenic Dyskinesia.

Members of family	Patient 1	Patient 2	Patient 3
Age (years)	46	14	12
Gender	male	male	male
Age of onset (years)	10	10	10
Clinical feature Dystonia	+	+	+
Chreoathetosis	-	+	+
Distribution Bilateral or alternating sides	+	Left side only	Left side only
Frequency	variable	4-5times/day	4-5times/day
Precipitating factor Sudden movement	+	+	+
Anxiety	+	-	+
EEG/MRI	--	AbN/N	AbN/N
Epilepsy	-	-	-
AED	CBZ	CBZ	CBZ
Response to AED	--	Effective	Effective

EEG: eletroencephalogram

MRI: magnetic resonance image

AED: Antiepileptic drug CBZ: carbamazepine

AbN: abnormal

N: normal

### 2.2. Mutation Analysis

After written consent, genomic DNA was extracted from peripheral leukocytes by AxyPrep Blood Genomic DNA kit (Axygen Biosciences, Union City, CA). Our mutation analysis of *PRRT2* used direct sequencing after PCR amplification (primer sequences available on request) in three patients, with amplified fragments directly sequenced by BigDye Terminator v3.1 Cycle Sequencing kit (Applied Biosystems, Foster City, CA) and run on ABI PRISM 3130_l Genetic Analyzer (Applied Biosystems). Results show p.R217Pfs*8 (c.649_650insC) in three patients (Fig. B).

**Fig. 1:**
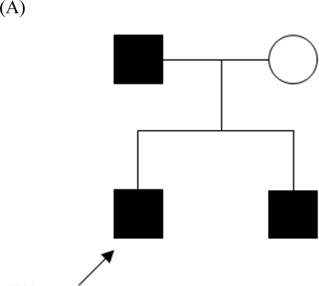

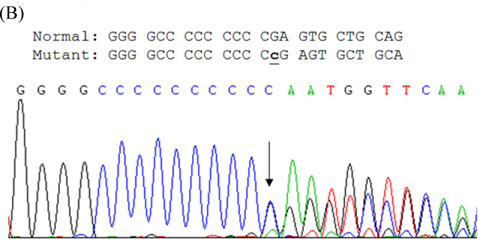
(A) Pedigree of PKC/PKD Taiwanese family: persons designated by sex, disease status (filled symbols represent patients, open symbols normal persons). Index case indicated by arrow. (B) Sequencing results of mutation in PRRT2 gene of index case. Arrow indicates one-base C inserted at nucleotide 650 (c.650insC), causing protein translation shift and stopping after seventh residue.

## 3. Discussion

PKD is the most common form of paroxysmal dyskinesia. Clinical features of our patients resembled cases reported previously: e.g., precipitating factors, patterns of attack, age of onset [1, 2, 4, 5, 6, 16]. Overall, family members had clinical features similar to those of individuals in literature. Controversial surrounds pathophysiology of involuntary movements. It is still uncertain whether symptoms relate to epileptic seizure or are dysfunction of basal ganglia [17, 18, 19]. Some propose this disorder as epileptic syndrome, based on prodromata preceding attacks, as well as its response to anticonvulsants. Interictal EEG abnormalities of reported PKD cases include sporadic epileptic discharge or slow rhythm [7, 8, 9]. None of our recordings showed any such abnormalities. Also, consciousness during attacks was always preserved.

Patients with PKD attacks seem to respond well to anticonvulsants: lamotrigine, phenytoin, valproate, oxacarbazepine, and especially CBZ [5, 6, 20, 21, 22], whose mechanism is blockade of ion conduction through voltagedependent ion channels of the neuron. Physiology of PKD is still uncertain, but ion channelopathy is considered; prior studies reported PKD patients sensitive to ion channel blockers like CBZ [6, 21]. CBZ is widely used, being inexpensive and broad-spectrum in seizure controls; we found 100mg/day effectively controlling PKD. Therapeutic dose ranged from 1.5 to 2.0 mg/kg/day, lower than that in seizure control. No patients treated with CBZ had intellectual impairment or decline of school performance during follow-up.

Seventeen *PRRT2* mutations were recently identified in patients with familial or apparently sporadic PKD/IC from several ethnic groups [11-15, 23], the vast majority (11/17) premature termination or frameshift mutation resulting in truncation of PRRT protein, the rest missense mutations. Interestingly, p.R217P*8 arose in about 80% of PKD patients with *PRRT2* mutations [11-15, 23]. All identified families displayed autosomally dominant inheritance. Function of *PRRT2* protein is poorly characterized. Recent *in vivo* and *in vitro* studies demonstrated that *PRRT2* is highly expressed in the developing nervous system and localized on cell membrane, predominantly in axons [11, 14]. It is not surprising that truncating mutations can significantly reduce protein expression and/or cause loss of transmembrane property, which conceivably impairs *PRRT2* protein function [11, 14]. This study highlights frequency of *PRRT2* mutations in a Taiwanese cohort with idiopathic PKD. For this benign neurological condition, low dose of CBZ is adequate to provide good control.

## Acknowledgments

This study was supported in part by the China Medical University Hospital (grant number DMR-103-034).
